# Drivers of diet selection of critically endangered Western Derby eland during the food shortage period within conservation breeding in Senegal

**DOI:** 10.1038/s41598-019-45035-z

**Published:** 2019-06-18

**Authors:** Pavla Hejcmanová, Magdalena Miřejovská, Petr Homolka, Michal Hejcman

**Affiliations:** 1 Department of Animal Science and Food Processing, Faculty of Tropical AgriSciences, Czech University of Life Sciences Prague, Kamýcká 129, CZ 165 00 Prague 6 - Suchdol, Czech Republic; 2 Department of Microbiology, Nutrition and Dietetics, Faculty of Agrobiology, Food and Natural Resources, Czech University of Life Sciences Prague, Kamýcká 129, CZ 165 00 Prague 6 - Suchdol, Czech Republic; 3 Institute of Animal Science, Přátelství 815, CZ 104 00 Prague 22 - Uhříněves, Czech Republic; 4 Department of Ecology, Faculty of Environmental Sciences, Czech University of Life Sciences Prague, Kamýcká 129, CZ 165 00 Prague 6 - Suchdol, Czech Republic

**Keywords:** Conservation biology, Tropical ecology, Animal behaviour

## Abstract

Browsers represent a challenge for breeding facilities because of their sensitivity to nutritional management. Western Derby eland (*Tautrotragus derbianus derbianus*, WDE) is a large browsing antelope with a very diverse diet. Because of its critically endangered status, a small WDE population is kept for conservation purposes in the fenced Fathala reserve (Senegal) and during the critical, hot dry season, the animals are offered supplementary *Acacia albida* pods. We aimed to identify which woody plant species were preferentially selected/avoided by WDE during the period of food shortage, which plant nutritional properties were drivers of animals’ diet selection, and how this selectivity was affected by supplemental feed. The animals were selective for certain plant species, most for *Piliostigma thonningi* pods. Preferences decreased with a feed supplement, while avoidances remained intact. Diet selection was connected with chemical traits, mostly by negative correlations to N, Mg, Ca and hemicellulose, which disappeared or were weaker when supplemental feed was offered. Our findings indicate that large browsers during periods of food shortage must cope with inappropriate chemical composition in regard to nutrition and seek to alleviate them not only by diversification of plant species in the diet, but also by adjusting chemical diet quality as a whole.

## Introduction

Western Derby eland (*Tautrotragus derbianus derbianus*, WDE) is a large, West African savannah dwelling antelope, listed by IUCN Antelope Specialist Group as critically endangered^1^. The current area of WDE distribution is limited to the Niokolo Koba national park in south-east Senegal, and there is an established semi-captive population in a conservation breeding program in two managed wildlife reserves in Senegal^2^. One of the keys for the animals’ health and breeding fitness is to ensure an optimal diet. WDE has been observed to consume predominantly leaves, flowers, and fruits of woody plants in the wild and in both reserves^3,4^ and can be therefore classified as a browser.

Browsers represent a challenge for conservation breeding facilities as they are very sensitive in their nutritional management, particularly to low fibre contents resulting from a rejection of commonly available fibre sources, and consequently they have a high prevalence of gastrointestinal upsets^5^. Browsers’ diets typically contain high lignin concentrations and are therefore less digestible than grasses^6,7^. Leaves of woody plants often contain secondary plant compounds, which may act as further digestibility reducers and feeding deterrents. According to optimal feeding theory, herbivores prefer a diet of high protein content, with a high digestible or metabolisable energy content and a low content of anti-nutritional or toxic compounds. Interplay between chemical compounds in forage plants might, however, lead to a complicated choice of diet^8,9^ with a trade-off between nutrient acquisition and avoidance of plant constituents which might be toxic for animals or that limit forage digestibility^10^. Highly diverse herbivore diets, as in the case of WDE in the wild^11^, considered an evolutionary adaptation to varied contents of nutrients accompanied by toxins, imply preferences for and avoidances of particular plant species^12^. Animals might select diets with a higher content of antinutritional or imbalanced compounds as a consequence of selection for nutrients in plants which contain both type of compounds together^13,14^, especially in critical periods of food supply shortage and decrease of nutrient contents below optimal levels^15^.

To minimize nutritional stress of WDEs in breeding facilities within reserves, a supplemental feed is provided to animals during the hot dry season. This can have consequences for management, because supplemental feeding has the strong potential to change the behaviour of animals^4,16^. We therefore expected that it may also interfere in the selection of diet. Our aim was to identify the determinants of diet selection of WDEs in the conservation program in the Fathala wildlife reserve in Senegal during the hot dry season, the most critical period of food shortage. First, we explored which plant species were available in the enclosure, which were preferentially selected and avoided by WDE, and how the selection for plant species was affected by supplemental feed. Then, we investigated the interplay among chemical properties of plants, i.e. content of macro-elements and fibre fractions, and their effect on diet selection.

## Results

**Figure 1:**
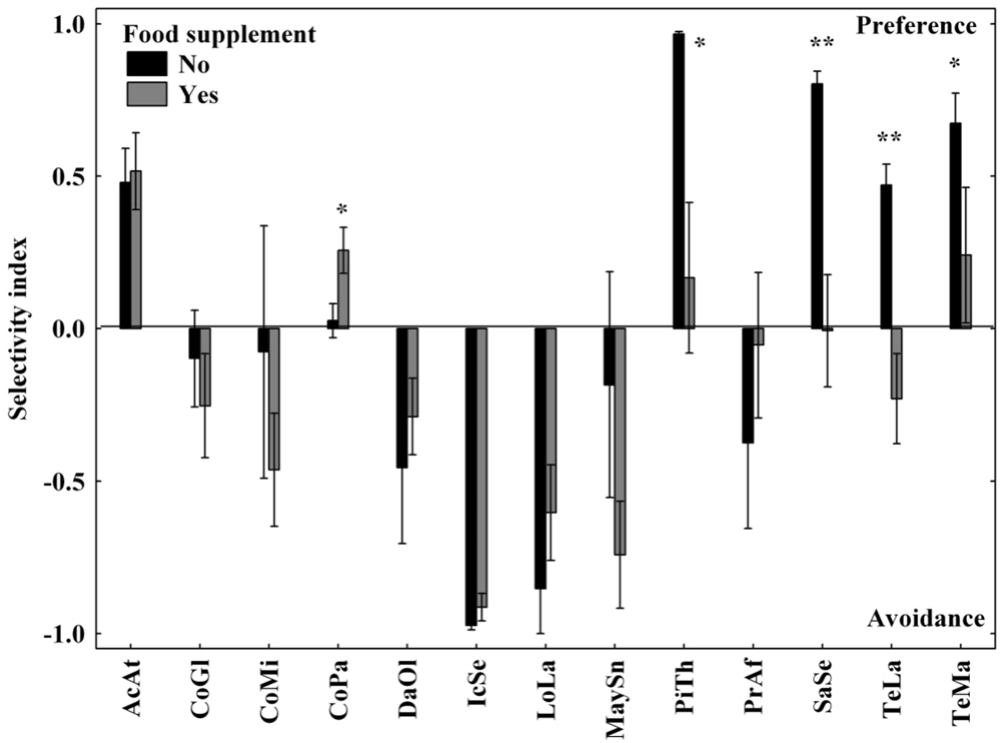
Selectivity of Western Derby elands for particular plant species with and without offered feed supplement (pods of *A. albida*) in the Fathala reserve during April and May 2008. The error bars indicate SE. The effect of supplementation is indicated by *(*P* < 0.05) or **(*P* < 0.01). For abbreviations, see Table 1.

The most abundant plant biomass in the enclosure was dry herbage (mean cover 18.4% ± 5.6%SE), and only a small portion was live herbage (mean cover 1.25% ± 0.3%SE). The most abundant live grass was *Andropogon gayanus* (mean cover 0.92% ± 0.3%SE). Thirty six woody plant species were identified, of which the most abundant were *Icacina senegalensis*, *Daniellia oliveri*, and *Combretum glutinosum*. Thirty two woody plant species were part of WDEs’ natural diet in the FR. *Anacardium occidentale*, *Ficus* sp., *Ozoroa insignis*, and *Parkia biglobosa* were not browsed by WDE. Cover of 10 woody plant species of interest is given in Table 1. Animals selected some plant species more than others (*F*_(12,234)_ = 10.85, *P* < 0.001). *Acacia ataxacantha*, *Piliostigma thonningii*, and *Terminalia macroptera* were the most preferred and *Icacina senegalensis*, *Lonchocarpus laxiflorus*, and *Maytenus senegalensis* were the most avoided species, respectively. Other plant species were browsed in proportion to their biomass availability in the enclosure (Fig. 1). *Saba senegalensis*, *Terminalia laxiflora*, *T. macroptera*, and pods of *P. thonningii* were browsed selectively without feed supplement, whereas when the supplemental feed was offered, the preference decreased and they were browsed in proportion to their availability. *Combretum paniculatum* was the only plant species selected more with feed supplement. For other plant species, there was no significant effect of supplementation on selectivity (Fig. 1).

**Table 1:** Concentration (mean ± standard error of mean) of fibre fractions and ash in biomass of studied woody plant species (N = 4 per species), the mean selectivity index (S_i_) (without and with feed supplement together, N = 200 observation events), and mean biomass cover (in%) of woody plant species in the WDEs enclosure in the Fathala reserve (N = 19 vegetation plots) during April and May 2008.

Species	Plant code	NDF (g kg^−1^)	ADF (g kg^−1^)	ADL (g kg^−1^)	Cellulose (g kg^−1^)	Hemicellulose (g kg^−1^)	Ash (g kg^−1^)	Si	Mean cover (%)
*Acacia ataxacantha*	AcAt	427 ± 53^ab^	374 ± 26^abcd^	264 ± 15^b^	110 ± 36^a^	54 ± 30^abc^	80 ± 16^abc^	0.51 ± 0.09^a^	2.46 ± 0.3
*Combretum glutinosum*	CoGl	393 ± 9^abc^	321 ± 8^ce^	61 ± 4^d^	260 ± 6^d^	72 ± 11^abc^	45 ± 6^ad^	−0.21 ± 0.13^bc^	6.97 ± 1.4
*Combretum micranthum*	CoMi	279 ± 8^c^	257 ± 7^e^	133 ± 12^a^	124 ± 17^ab^	22 ± 6^ab^	75 ± 7^bc^	−0.35 ± 0.18^cd^	0.68 ± 0.1
*Combretum paniculatum*	CoPa	440 ± 27^ab^	422 ± 19^ab^	270 ± 13^b^	151 ± 27^abc^	18 ± 8^ab^	71 ± 13^abc^	0.19 ± 0.06^abc^	6.16 ± 2.2
*Daniellia oliveri*	DaOl	496 ± 34^a^	415 ± 13^ab^	227 ± 14^bc^	189 ± 17^abcd^	80 ± 22^bc^	82 ± 4^abc^	−0.34 ± 0.11^cd^	7.35 ± 1.4
*Icacina senegalensis*	IcSe	472 ± 11^a^	357 ± 7^acd^	149 ± 8^a^	207 ± 2^bcd^	116 ± 7^c^	103 ± 10c	−0.93 ± 0.03^d^	9.78 ± 2.3
*Piliostigma thonningii* pods	PiTh	447 ± 13^ab^	419 ± 6^ab^	181 ± 14^ac^	238 ± 13^d^	29 ± 14^ab^	45 ± 2^ad^	0.41 ± 0.19^a^	0.11 ± 0.02
*Saba senegalensis*	SaSe	437 ± 35^ab^	400 ± 21^abd^	266 ± 29^b^	134 ± 10^abc^	37 ± 16^ab^	75 ± 2^abc^	0.24 ± 0.15^abc^	2.63 ± 0.4
*Terminalia laxiflora*	TeLa	461 ± 13^ab^	429 ± 4^b^	191 ± 11^ac^	238 ± 8^d^	32 ± 13^ab^	49 ± 4^abd^	−0.02 ± 0.13^abc^	3.00 ± 1.2
*Terminalia macroptera*	TeMa	345 ± 15^bc^	339 ± 11 ^cd^	129 ± 18^ad^	209 ± 11^cd^	6 ± 4^a^	85 ± 1^bc^	0.37 ± 0.16^ab^	0.84 ± 0.2
*Acacia albida* (pods)	AcAl	392 ± 2^abc^	385 ± 6^abcd^	143 ± 2^a^	242 ± 4^d^	7 ± 4^a^	31^d^	NA	NA
Optimum range for cattle^34,35^		330–450	190–300	max. 80					

Calculated by one-way ANOVA, differences among species for all chemical properties were significant (*P* < 0.01). Using Tukey *post-hoc* comparison test, species with the same letter were not significantly different.

Abbreviations: NDF - neutral detergent fibre, ADF - acid detergent fibre, ADL - acid detergent lignin.

There were significant differences in nutrient and macro-element (N, P, K, Na, Ca and Mg) concentrations between several of plant species investigated (all *P* < 0.01; Tables 1 and 2).

**Table 2:** Concentration (mean ± standard error of mean) of N, P, K, Na, Ca, Mg and N:P, Ca:P ratios in biomass of studied species (N = 4 per species) collected in the Fathala reserve during April and May 2008.

Species	Plant code	N (g kg^−1^)	P (g kg^−1^)	K (g kg^−1^)	Na (g kg^−1^)	Ca (g kg^−1^)	Mg (g kg^−1^)	N:P ratio	Ca:P ratio
*Acacia ataxacantha*	AcAt	20.8 ± 2.8^abc^	1.2 ± 0.17^ab^	3.5 ± 0.6^a^	0.32 ± 0.05^ef^	20.0 ± 5.0^ac^	2.3 ± 0.1^ac^	18.4 ± 1.8^ab^	20.2 ± 6.7^a^
*Combretum glutinosum*	CoGl	18.0 ± 1.0^abd^	1.3 ± 0.13^ab^	6.4 ± 0.1^abc^	0.19 ± 0.02^abc^	8.3 ± 1.5^abd^	2.8 ± 0.4^abc^	13.8 ± 0.8^a^	6.7 ± 1.7^abc^
*Combretum micranthum*	CoMi	22.7 ± 1.1^bc^	1.0 ± 0.04^a^	8.0 ± 1.0^abc^	0.36 ± 0.02 ^f^	18.1 ± 2.5^abc^	3.3 ± 0.3^abc^	22.8 ± 1.1^b^	18.4 ± 2.9^ab^
*Combretum paniculatum*	CoPa	24.3 ± 2.2^bc^	1.7 ± 0.11^ab^	5.9 ± 0.4^abc^	0.21 ± 0.01^abcd^	16.8 ± 4.8^abc^	3.9 ± 0.9^bc^	14.1 ± 0.8^a^	10.0 ± 3.0^abc^
*Daniellia oliveri*	DaOl	19.9 ± 0.5^abc^	1.3 ± 0.11^ab^	4.6 ± 0.5^ab^	0.23 ± 0.01^bcde^	16.6 ± 1.3^abc^	4.8 ± 0.2^bd^	15.6 ± 1.3^a^	13.2 ± 1.9^abc^
*Icacina senegalensis*	IcSe	25.7 ± 1.4^c^	1.4 ± 0.04^ab^	5.9 ± 0.4^abc^	0.20 ± 0.01^abc^	26.7 ± 3.9^c^	6.3 ± 0.7^d^	18.42 ± 1.0^ab^	19.3 ± 3.1^ac^
*Piliostigma thonningii* (pods)	PiTh	11.7 ± 1.3^d^	1.8 ± 0.13^b^	13.2 ± 0.8^d^	0.13 ± 0.01^a^	5.9 ± 0.4^bd^	1.3 ± 0.1^a^	6.47 ± 0.8^c^	3.3 ± 0.3^bc^
*Saba senegalensis*	SaSe	19.2 ± 0.3^abc^	1.3 ± 0.03^ab^	8.3 ± 1.0^bc^	0.26 ± 0.02^cde^	15.8 ± 0.8^abcd^	3.4 ± 0.3^abc^	15.08 ± 0.5^a^	12.4 ± 0.9^abc^
*Terminalia laxiflora*	TeLa	15.4 ± 0.1^ad^	1.1 ± 0.06^ab^	4.9 ± 0.5^ab^	0.22 ± 0.01^abcd^	9.7 ± 0.8^abd^	2.8 ± 0.2^abc^	13.86 ± 0.8^a^	8.6 ± 0.3^abc^
*Terminalia macroptera*	TeMa	14.1 ± 2.3^ad^	1.3 ± 0.39^ab^	5.9 ± 2.4^abc^	0.30 ± 0.02^def^	19.4 ± 1.7^ac^	4.7 ± 0.5^bd^	13.11 ± 1.9^a^	21.8 ± 7.0^a^
*Acacia albida* (pods)	AcAl	21.2^abc^	1.4^ab^	10.3 ^cd^	0.15^ab^	3.1^d^	1.4^a^	14.89^a^	2.2^b^
Optimum range for cattle^34–36^		19.2–25.6	2.3–4.4	5–10	0.6–1.2	2.9–11	1.5–3.5	5–10	1–2

Calculated by one-way ANOVA, differences among species for all chemical properties were significant (*P* < 0.01). Using Tukey *post-hoc* comparison test, species with the same letter were not significantly different.

**Figure 2:**
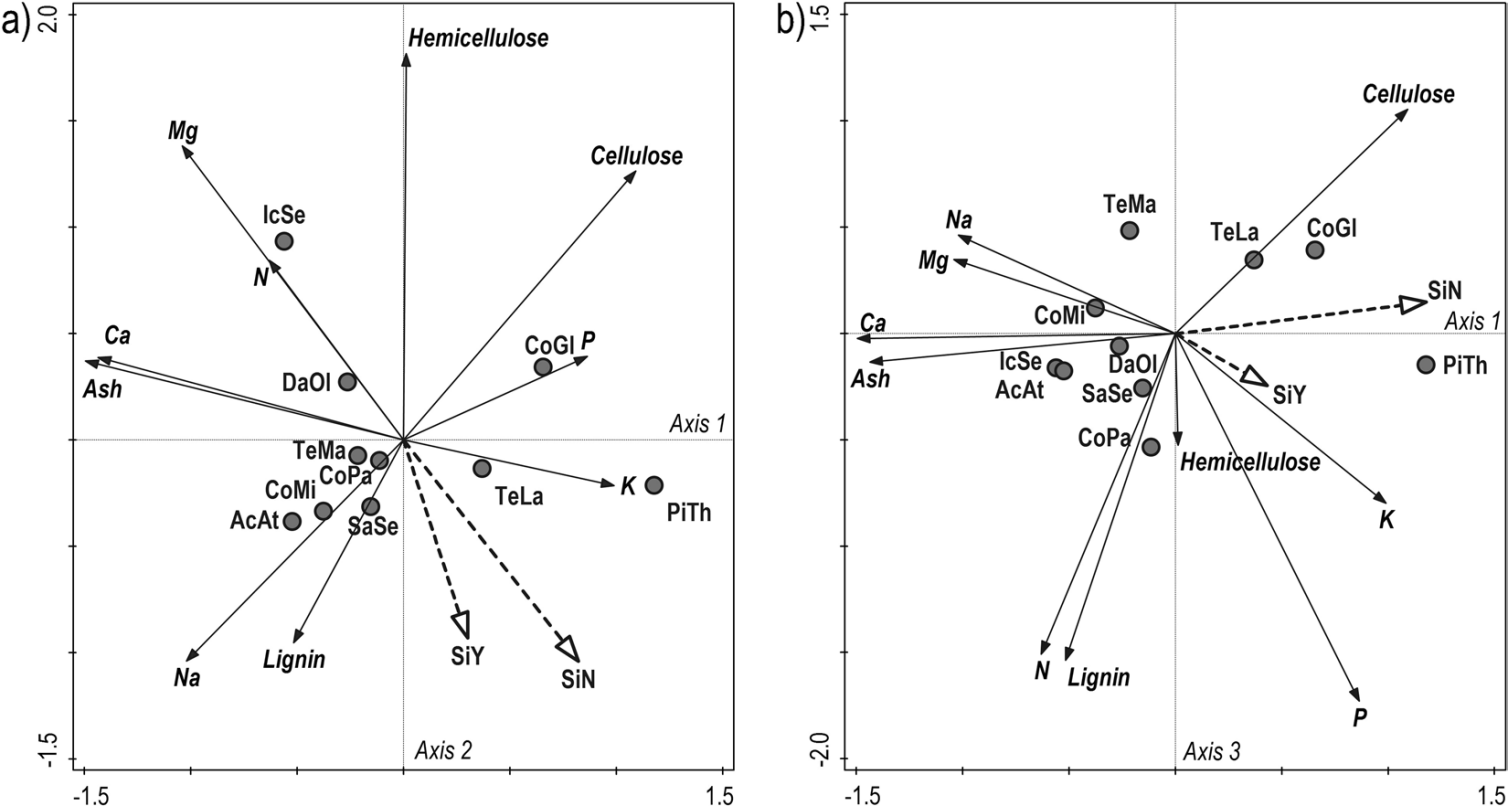
Ordination diagram showing mutual relationships of chemical traits in ten plant species which are part of the Western Derby elands’ diet in the Fathala reserve, complemented by projection of the level of selectivity (selectivity index) without (SiN) and with (SiY) feed supplement. For plant species codes see Table 1. Numbers following species codes refer to the number of the sample.

Regarding the relationships of all chemical traits together, the first axis of PCA explained 37% of data variability and represented content of Ca, K, and ash. The second axis explained 19% of data variability and represented mainly the content of hemicellulose and partly Mg. The third axis explained 15% of data variability and represented the content of N, P and lignin (Fig. 2). All (four) axes together explained 81% of data variability. Regarding correlations of plant species projected passively in the PCA plot as supplementary variables towards axes and nutrients, *P. thonningii*, was correlated to the first axis, and together with *T. laxiflora* and *C. glutinosum* were positively related to K and P concentration and to cellulose, negatively related to concentration of Ca, Mg and ash; *I. senegalensis* was correlated to the second axis, and together with *Danielia oliveri* was positively related to content of hemicellulose and N and Mg concentration; *C. paniculatum, C. micranthum, T. macroptera*, and *T. laxiflora* were correlated with the third axis and appeared negatively related to lignin, N and P (Fig. 2). Diet selectivity without and with feed supplement showed similar relationships to plant species and their chemical properties, both correlated with second axis, negatively to hemicellulose, and to Ca, Mg, and ash.

When the data set of selectivity were sorted according to feed supplement and pooled across plant species, the relationships between selectivity index and chemical traits separately were obtained (Table 3). When animals browsed on the natural food, the selectivity index was negatively correlated to N, Ca, Mg, ash and hemicellulose and positively to K; while when feed supplement was offered, the selectivity index was negatively correlated to N, Mg, and hemicellulose only, and positively to ADF and ADL.

**Table 3:** The Pearson’s correlation between the selectivity index (S_i_) and chemical properties of plant species when they browsed on natural vegetation without the feed supplement and when they received it.

Selectivity index	N	P	K	Na	Ca	Mg	Ash	NDF	ADF	ADL	Cellulose	Hemicellulose
Without feed supplement	−0.624*	0.098	0.358*	0.032	−0.450*	−0.603*	−0.418*	−0.137	0.223	0.205	−0.037	−0.630*
With feed supplement	−0.231*	0.129	0.012	0.064	−0.154	−0.314*	−0.146	−0.026	0.184*	0.264*	−0.155	−0.353*

*p < 0.05.

## Discussion

Selectivity of WDEs for plant species in the FR varied and were mostly similar to preferences recorded in wild and domestic animals elsewhere, see e.g. preferences of *A. ataxacantha*^17^ or *Terminalia* spp.^18,19^. We however noted that regarding *Terminalia* spp. the animals did not feed only on leaves directly from trees, but they spent also a considerable time to ingest leaves from the ground, i.e. mostly dry, senescent leaves. This observation brings an interesting new view on the use of food resources for such large browsing herbivore in the whole range of available and reachable space between 0 and 2 m of height. Pods of *P. thonningii* preferentially selected by WDE were reported as highly selected also by other large savannah herbivores^19^ and by livestock^20^ and are available at local markets as a local “commercial” feed supplement for livestock in West African countries. *P. thonningii* had mature pods in the investigated hot dry season with food shortage. Because these highly preferred trees were not too abundant and grew in groups, i.e. not regularly scattered in the reserve, their spatial distribution has the potential to shift the habitat use of the animals. Drivers for specific plant species preferences in the diet could be a combination of nutritive values together with antibacterial^21^ and/or anti-inflammatory^22^ properties. On the other hand, clear avoidance of *I. senegalensis, L. laxiflorus* and *M. senegalensis* by WDEs could be related most likely to the high content of toxic compounds, e.g. terpenoids, alkaloids, and/or phenolic acids^23^.

Plant species preferentially selected by WDEs in their diet did not stand out for content of any macroelement, similar to what has been found in Nguni goats browsing in a sub-humid savannah^24,25^. They were in the lower range of values for N content of all measured plants, and contained less hemicellulose and more cellulose in comparison to other plant species. Hemicellulose and cellulose showed similar values as supplementary and highly preferred *A. albida* pods. The pattern of chemical traits of avoided plant species displayed higher content of Mg and hemicellulose, and higher range of values of N content. WDEs’ selectivity (S_i_) did not show any pronounced positive correlation to any element. On the contrary, the selectivity index was conspicuously and relatively strongly negatively correlated to N, Ca, Mg, ash and hemicellulose. The WDEs’ negative selectivity pattern for high N concentration in plants appeared likely because high N concentrations indicated not only protein-origin N, but also an important proportion of non-protein N contained in toxic compounds^23^ or simultaneous high content of condensed tannins constraining the digestibility of plants^26^. Neither tannins, nor N-based secondary metabolites were, however, measured in our study and will require special attention in future studies on the WDEs. Regarding Mg, Ca and ash, woody plants in the Fathala reserve showed rather high concentrations in their leaves, largely exceeding optima for adequate nutrition for ruminants, which may lead animals to reduce their intake. Revealed strong negative correlation of WDEs diet selection with content of hemicellulose seems to be rather a concurrent effect than a real driver of diet selection. Highly palatable woody plants attractive for herbivores, however, evolve various herbivore-avoidance strategies against herbivores which includes also a chemical defence^27^. Then, the animals respond by avoiding them or diminishing their contents in their diet items, together with attractive and nutritive compounds. This suggests that the WDEs diet selection would be driven not in favour of, but rather in order to avoid or alleviate specific chemical traits in their diet (high contents of Mg, Ca and ash), i.e. the animals selected plant species with lower concentrations of these elements to compile an appropriate combination of nutrients in order to cope with limited resources available in the African savanna in extreme periods. Apart from metabolic demands, selection of the diet is usually considered to be driven by deficiencies of particular nutrients in animal’s immediate environment^8,28^. Nutritive value of browse plants in the FR might not be, however, comparable with studies from different regions because nutrient deficiencies are site-specific and nutritive value of foliage of the same plant species highly vary. Nonetheless, our results indicate that regarding the content of macroelements and fibre fractions in plants relative to nutritive value for large herbivores, the overall nutritive value of WDE food resources in the FR is lower in comparison to other studies^9,18,29,30^, appears to be unfavourable and animals respond not only by diversification of diet, but also by a selective foraging strategy.

The supplemental feed had an effect on the WDE diet selectivity only for plant species which were preferentially selected, and the supplemental feed diminished the preference, with the only exception of *C. paniculatum*. With supplemental feed, negative correlations of nutrient concentrations with feeding selectivity weakened. Both results together indicate that the supplemental feed diminished pressures connected with the natural diet, which could be both amount of food and chemical composition. We suggest that supplemental *A. albida* pods offered to animals satisfied WDEs’ need to fill up the ingestive tract by biomass with adequate nutritive value, therefore highly preferred plants *S. senegalensis*, *T. laxiflora*, *T. macroptera*, and pods of *P. thonningii* were not searched any more under any selective pressure. We point out that highly preferred pods of *P. thonningii* had similar nutrient composition as *A. albida* pods and are therefore mutually substitutable. Real abundance of *P. thonningii* in the fenced area is, however, scarce and cannot satisfy the needs of animals in the reserve and makes thus the supplementation in the FR an important factor for WDE to cope with natural dietary conditions during the constrained period.

## Materials and Methods

The study was conducted in the Fathala reserve (FR), a fenced wildlife reserve in Senegal (13°39′N; 16°27′W). The climate has a seasonal character with a dry season from November to May and a rainy season from June to October, with annual mean precipitation of 1022 mm and the mean annual temperature 26 °C. The area belongs to the Sudanese/ Sudano-Guinean savannah and contains three vegetation types: wooded grassland, woodland and the transitional woodland with dominants of *Acacia macrostachya*, several species of the *Combretum* genus*, Piliostigma thonningii, Prosopis africana*, *Pterocarpus erinaceus, Terminalia avicennoides*, and *T. macroptera*^31^. The study was performed in the 70 ha enclosure within the FR maintained for purposes of WDE conservation breeding on a herd of six animals (one male and five females), the only herd for reproduction held in the reserve in 2008. We collected data on plant species availability and animal behaviour in April and May 2008.

To determine the animals’ selectivity for particular plant species, we used Jacobs’ selectivity index^32^: S_i_ = c_i _− a_i_/c_i_ + a_i_ − 2c_i_a_i_, where a_i_ represents the proportion of each plant species available as potential forage in the enclosure (values ranged from 0 to 1) and c_i_ represents the proportion of particular plant species in the animal diet as forage used by animal (values ranged from 0 to 1). To determine the proportion of each plant species (a_i_) we used estimated percentage cover for each plant species as a relative indicator of available forage. We set a regular grid of 19 square plots of 200 m^2^ at a distance of 200 m apart over the enclosure. In each plot we identified all woody plant species and we estimated percentage cover of leaf biomass for each plant species^30^ till the height of 2.5 m (as being available for the WDE, pers. obs.).

We derived the value of c_i_ from the time that each observed animal spent browsing on each plant species during one observation session, as this parameter was relevant to the animal decision to browse on the plant. To determine this, we observed six (i.e. all present) WDE individuals in the enclosure directly at a distance of 15 to 30 m, using binoculars when necessary. The animals were of the same age (2–3 years old) and all in non-reproductive state. One observation session was made by one observer continuously during 10 h of daylight (mostly from 7:00 to 17:00) on one focal animal. Total study consisted in repeated observations during 22 observation days (one individual per day). We repeated observation at least three times for each animal in a randomized time schedule. We recorded browsed plant species and time spent browsing on it and calculated c_i_ for each animal observation session. From these parameters we calculated Jacobs’ index of selectivity, S_i_, of each individual animal in one observation session for ten plant species (Table 1), which were relatively abundant in the enclosure and formed part of the WDE diet^4^. Values of S_i_ varied from −1 to 1 (S_i_ = −1 means never browsed; S_i_ = 1 exclusively browsed; and S_i_ = 0 means that the species was browsed in proportion to its availability).

We calculated the selectivity index for the plant species in the animal diet (1) with naturally available food resources within the enclosure in the hot dry season, and (2) when food was increased by supplementary feed (for all animals together 20 kg of fresh mature pods of *Acacia albida*, a standard, commonly used as a supplement for livestock in Senegal) offered to animals in the morning at a predetermined place in the enclosure. The pods were presented in a line to enable the access to all animals and to minimize competition for feed. Situations with and without feed supplement were equally represented over 22 observation days, alternating each three subsequent days (all animals were observed twice in each situation, except two individuals).

Ten plant species (Table 1) selected for determination of diet S_i_ were analysed for their chemical properties. We collected leaves of nine plant species and one sample of fruits (pods of *Piliostigma thonningii*), for each plant four independent samples. Biomass samples, including a single sample of the supplementary feed (*A. albida* pods), were oven-dried at 60 °C for 72 h and milled. Concentration of macro-elements (N, P, K, Na, Ca, Mg) and the content of ash, neutral detergent (NDF), acid detergent fibre (ADF) and acid detergent lignin (ADL) were determined. The N concentration was determined by combustion (950 °C) using a LECO TruSpec. The concentrations of P, K, Na, Ca and Mg in the biomass samples were determined by ICP-OES (IRIS Intrepid II XSP Duo) after a microwave-assisted dissolution in HNO_3_ and HCl (ratio 6:1). Ash content was obtained by burning the samples in a furnace at 550 °C to constant weight. Ash-free NDF, ADF and ADL contents were determined by standard methods of AOAC^33^, content of hemicellulose and cellulose was determined by subtractions NDF-ADF and ADF-ADL, respectively.

Data (selectivity index S_i_, concentrations of macro-elements and fibre fractions) met the assumption of normal distribution (tested by Kolmogorov-Smirnov test). Foraging behaviour was recorded on animals repeatedly and these data were considered as repeated measures. To test the degree of selectivity (S_i_) of WDE for a particular plant species and the effect of supplemental feed on selectivity, we applied a GLMM with categorical predictors ‘individual animal’ (as a random factor), ‘plant species’, ‘supplemental feed’, interaction ‘plant species*supplemental feed’. Significant effects (α = 0.05) were followed by *post hoc* comparison Tukey HSD test. At the plant species level, we used Student’s t-tests to test the difference between S_i_ for each plant species separately, without and with the supplementary feed. We applied ANOVA followed by *post hoc* comparison Tukey’s HSD test to identify differences in chemical properties among particular plant species. To test the relationships between chemical properties and S_i_, we applied separate Pearson’s correlation analyses for each chemical trait without and with supplemental feed. All univariate analyses were carried out at significance level α = 0.05 in the STATISTICA package (StatSoft, Tulsa). To evaluate the interplay of chemical properties in plant species, we applied principal component analysis (PCA) with passive projection of plant species and S_i_ in the Canoco 5 program (www.canoco5.com).

## Data Availability

All data used in the study are available on request.
